# Supplementary material for: Resolving the full spectrum of human genome variation using Linked-Reads
Source: Genome Res. 2019 Apr;29(4):635–45. doi: 10.1101/gr.234443.118 (PMC6442396; doi:10.1101/gr.234443.118)
Supplement: Supplemental Material [file supp_gr.234443.118_Supplemental_longranger-2.2.zip › longranger-2.2.tar.bz2!/lr2.2-public/lib/stan/cmdstan/stan/doc/api/html/init__windowed__adapt_8hpp.html]

Stan: src/stan/common/init\_windowed\_adapt.hpp File Reference


|  |  |
| --- | --- |
|  | Stan  2.5.0  probability, sampling & optimization |


- Main Page
- Related Pages
- Namespaces
- Classes
- Files

- File List
- File Members

All Classes Namespaces Files Functions Variables Typedefs Enumerations Enumerator Friends Macros Pages

- src
- stan
- common

Namespaces |
Functions

init\_windowed\_adapt.hpp File Reference

`#include <stan/mcmc/base_mcmc.hpp>`  
`#include <stan/gm/arguments/categorical_argument.hpp>`  
`#include <stan/math/matrix/Eigen.hpp>`  
`#include <stan/common/init_adapt.hpp>`

Go to the source code of this file.

|  |  |
| --- | --- |
| Namespaces | |
|  | stan |
|  | Probability, optimization and sampling library. |
|  | |
|  | stan::common |
|  | |

|  |  |
| --- | --- |
| Functions | |
| template<class Sampler > | |
| bool | stan::common::init\_windowed\_adapt (stan::mcmc::base\_mcmc \*sampler, stan::gm::categorical\_argument \*adapt, unsigned int num\_warmup, const Eigen::VectorXd &cont\_params) |
|  | |


---

[ Stan Home Page ]

*© 2011–2014,
Stan Development Team.*
